# Supplementary material for: Prevalence of double heterozygotes of HbE and α-thal 1 (SEA) type in pregnancies and their partners that received antenatal care at Chiangrai Prachanukroh Hospital and reevaluated the cut-offs for differentiation
Source: PLoS One. 2025 Oct 7;20(10):e0333761. doi: 10.1371/journal.pone.0333761 (PMC12503262; doi:10.1371/journal.pone.0333761)
Supplement: S2 Methods — Laboratory methods. (DOCX) [file pone.0333761.s002.docx]

**Methods**

1. **Quantification analysis of hemoglobins**

**Capillary electrophoresis**

- Quantification analysis of hemoglobin fractions performed by an automated analyzer (Sebia, France) using the capillary electrophoresis method. This assay is performed on the hemolysate of whole blood samples collected in EDTA blood tubes. The hemoglobins, separated in silica capillaries, are directly and specifically detected at an absorbance wavelength of 415 nm, which specific to hemoglobins. Internal controls (normal and pathological controls) were used before starting the new analysis sequence. Direct detection at 415 nm in capillaries yields relative concentrations (percentages) of individual hemoglobin zones.

1. **Identification of Southeast Asian (SEA) type**

**Real-time PCR with melting curve analysis**

- Blood was collected in an EDTA tube, then extracted DNA from whole blood using DNA Mini Kit. After that identification of Southeast Asian (SEA) type by using Real Time LightCycle 1.5 PCR (Roche, Germany). With the help of this instrument, real-time PCR and extremely quick cycling can be carried out. Results can be quantified and analysed simultaneously by monitoring fluorescence during amplification. Melting curve analysis allows mutation detection.
