# Supplementary material for: Prevalence of double heterozygotes of HbE and α-thal 1 (SEA) type in pregnancies and their partners that received antenatal care at Chiangrai Prachanukroh Hospital and reevaluated the cut-offs for differentiation
Source: PLoS One. 2025 Oct 7;20(10):e0333761. doi: 10.1371/journal.pone.0333761 (PMC12503262; doi:10.1371/journal.pone.0333761)
Supplement: S4 Table — Data collected from pregnant women and their partners (stratified by gender). (DOCX) [file pone.0333761.s004.docx]

**S4 Table.** **Data collected from pregnant women and their partners (stratified by gender).**

| **Aged** | **Gender** | **RBC**  **(million/uL)** | **Hb**  **(g/dL)** | **Hct**  **(%)** | **MCV**  **(fL)** | **MCH**  **(pg)** | **MCHC**  **(g/dL)** | **RDW**  **(%)** | **HbE level (%)** | **Hemoglobin typing** | **Grouping** |
| --- | --- | --- | --- | --- | --- | --- | --- | --- | --- | --- | --- |
| 35 | M | 4.54 | 11.7 | 35 | 77.1 | 24.5 | 26.4 | 12.8 | 31.7 | Hb E-trait | E |
| 32 | M | 4.18 | 11.0 | 33 | 79 | 25.4 | 27.4 | 14.3 | 29.6 | Hb E-trait | E |
| 28 | M | 4.25 | 11.3 | 34 | 80 | 24.5 | 26.3 | 13.5 | 27.6 | Hb E-trait | E |
| 26 | M | 4.93 | 11.7 | 35 | 71 | 23 | 25 | 12.6 | 13.9 | Hb E-trait | SEA/E |
| 33 | M | 5.00 | 13.3 | 40 | 80 | 25.4 | 27.5 | 13.5 | 27 | Hb E-trait | E |
| 28 | M | 4.95 | 14.1 | 42.2 | 85.3 | 23.4 | 25.6 | 13.5 | 26.6 | Hb E-trait | E |
| 30 | M | 5.85 | 12.7 | 38 | 65 | 21.7 | 18.3 | 13.6 | 20.9 | Hb E-trait | SEA/E |
| 28 | M | 5.51 | 12.7 | 38 | 69 | 23 | 25.9 | 13.5 | 19.3 | Hb E-trait | SEA/E |
| 30 | M | 4.82 | 13.3 | 40 | 83 | 23.6 | 26.5 | 14.1 | 26.2 | Hb E-trait | E |
| 40 | M | 4.35 | 10.7 | 32 | 73.6 | 24.5 | 26.9 | 12.6 | 28.9 | Hb E-trait | E |
| 35 | M | 5.20 | 12.7 | 38 | 73.1 | 25.4 | 28.6 | 13.5 | 26.9 | Hb E-trait | E |
| 37 | M | 5.13 | 13.4 | 40.2 | 78.3 | 24.6 | 26.3 | 13.6 | 26.7 | Hb E-trait | E |
| 25 | M | 4.71 | 13.0 | 39 | 82.8 | 25.3 | 28.4 | 14.2 | 27.9 | Hb E-trait | E |
| 27 | M | 4.68 | 12.0 | 36 | 77 | 23.2 | 25.4 | 12.4 | 25.7 | Hb E-trait | E |
| 28 | M | 4.38 | 12.0 | 36 | 82.1 | 25.3 | 28.1 | 12.8 | 27.4 | Hb E-trait | E |
| 26 | M | 4.80 | 12.7 | 38 | 79.2 | 26.4 | 33.2 | 13.6 | 21.8 | Hb E-trait | E |
| 37 | M | 4.51 | 11.7 | 35 | 77.6 | 23.5 | 26.4 | 13.6 | 29 | Hb E-trait | E |
| 24 | M | 4.68 | 11.0 | 33 | 70.5 | 23.2 | 26.4 | 13.5 | 15.8 | Hb E-trait | SEA/E |
| 30 | M | 5.37 | 12.0 | 36 | 67 | 22.3 | 28.4 | 14.1 | 19 | Hb E-trait | SEA/E |
| 27 | M | 4.46 | 11.7 | 35 | 78.5 | 23.2 | 25.4 | 12.8 | 26.3 | Hb E-trait | E |
| 22 | M | 4.86 | 12.7 | 38 | 78.2 | 24.8 | 26.3 | 12.8 | 32.9 | Hb E-trait | E |
| 36 | M | 4.21 | 11.5 | 34.5 | 82 | 24.5 | 26.4 | 12.8 | 31.8 | Hb E-trait | E |
| 33 | M | 4.13 | 11.5 | 34.6 | 83.7 | 24.3 | 26.8 | 13.5 | 26 | Hb E-trait | E |
| 44 | M | 4.41 | 11.7 | 35 | 79 | 26.5 | 33.2 | 12.8 | 30.9 | Hb E-trait | E |
| 35 | M | 5.23 | 12.7 | 38.2 | 73.1 | 23.4 | 28.3 | 13.6 | 29.4 | Hb E-trait | E |
| 20 | M | 3.93 | 9.9 | 29.7 | 75.6 | 24.6 | 32.4 | 13.6 | 29.2 | Hb E-trait | E |
| 26 | M | 4.29 | 11.8 | 35.4 | 82.5 | 24.6 | 32.4 | 13.6 | 26.2 | Hb E-trait | E |
| 28 | M | 4.30 | 12.0 | 36 | 83.8 | 27.9 | 33.2 | 12.6 | 29.4 | Hb E-trait | E |
| 19 | M | 4.83 | 11.7 | 35 | 72.5 | 21.9 | 22.6 | 12.8 | 28.5 | Hb E-trait | E |
| 26 | M | 4.94 | 12.0 | 36 | 72.9 | 24.3 | 26.3 | 13.6 | 27.8 | Hb E-trait | E |
| 23 | M | 4.87 | 12.0 | 36.1 | 74.1 | 24.6 | 33.2 | 13.5 | 29.9 | Hb E-trait | E |
| 25 | M | 4.61 | 11.7 | 35.2 | 76.4 | 24.3 | 27.2 | 13.5 | 22.2 | Hb E-trait | E |
| 31 | M | 4.87 | 12.7 | 38 | 78 | 23.8 | 26.8 | 12.6 | 26.5 | Hb E-trait | E |
| 30 | M | 4.32 | 12.3 | 36.8 | 85.1 | 24.8 | 26.3 | 12.8 | 26.2 | Hb E-trait | E |
| 36 | M | 4.63 | 12.0 | 36.1 | 78 | 22.4 | 24.3 | 13.5 | 26.7 | Hb E-trait | E |
| 31 | M | 4.89 | 13.0 | 39 | 79.8 | 25.3 | 28.4 | 14.2 | 30.3 | Hb E-trait | E |
| 19 | M | 4.53 | 11.9 | 35.8 | 79 | 26.6 | 25.4 | 12.4 | 31.6 | Hb E-trait | E |
| 28 | M | 4.14 | 12.0 | 36 | 87 | 25.3 | 28.1 | 12.8 | 25.3 | Hb E-trait | SEA/E |
| 28 | M | 5.60 | 9.0 | 27 | 48 | 15.9 | 26.1 | 14.4 | 16.3 | Hb E-trait | SEA/E |
| 28 | M | 4.43 | 11.7 | 35 | 79 | 20.9 | 21.8 | 12.4 | 29.2 | Hb E-trait | E |
| 25 | M | 4.50 | 12.0 | 36 | 80 | 26.7 | 33.2 | 12.6 | 29.8 | Hb E-trait | E |
| 27 | M | 5.83 | 10.7 | 32 | 54.9 | 24.3 | 26.8 | 12.8 | 15.3 | Hb E-trait | SEA/E |
| 36 | M | 4.21 | 11.5 | 34.5 | 82 | 24.5 | 26.4 | 12.8 | 31.8 | Hb E-trait | E |
| 33 | M | 4.13 | 11.5 | 34.6 | 83.7 | 24.3 | 26.8 | 13.5 | 26 | Hb E-trait | E |
| 44 | M | 4.41 | 11.7 | 35 | 79.3 | 24.9 | 26.3 | 12.8 | 30.9 | Hb E-trait | E |
| 35 | M | 5.23 | 12.7 | 38.2 | 73.1 | 23.4 | 28.3 | 13.6 | 29.4 | Hb E-trait | E |
| 22 | M | 3.90 | 9.9 | 29.7 | 75.6 | 24.6 | 32.5 | 14.5 | 29.2 | Hb E-trait | E |
| 26 | M | 4.29 | 11.8 | 35.4 | 82.5 | 24.6 | 32.4 | 13.6 | 26.2 | Hb E-trait | E |
| 28 | M | 4.30 | 12.0 | 36 | 83.8 | 27.9 | 33.2 | 12.6 | 29.4 | Hb E-trait | E |
| 30 | M | 4.68 | 11.0 | 33 | 70.5 | 23.2 | 26.4 | 13.5 | 26.2 | Hb E-trait | E |
| 19 | M | 4.83 | 11.7 | 35 | 72.5 | 21.9 | 22.6 | 12.8 | 28.5 | Hb E-trait | E |
| 26 | M | 4.94 | 12.0 | 36 | 72.9 | 24.3 | 26.3 | 13.6 | 27.8 | Hb E-trait | E |
| 23 | M | 4.87 | 12.0 | 36.1 | 74.1 | 24.6 | 33.2 | 13.5 | 29.9 | Hb E-trait | E |
| 25 | M | 4.61 | 11.7 | 35.2 | 76.4 | 24.3 | 27.2 | 13.5 | 22.2 | Hb E-trait | E |
| 29 | M | 4.27 | 11.7 | 35.2 | 82.4 | 25.3 | 28.3 | 14.2 | 26.4 | Hb E-trait | E |
| 31 | M | 4.87 | 12.7 | 38 | 78 | 23.8 | 26.8 | 12.6 | 26.5 | Hb E-trait | E |
| 30 | M | 4.32 | 12.3 | 36.8 | 85.1 | 24.8 | 26.3 | 12.8 | 26.2 | Hb E-trait | E |
| 33 | M | 4.21 | 11.5 | 34.5 | 82 | 24.5 | 26.4 | 12.8 | 31.8 | Hb E-trait | E |
| 33 | M | 4.13 | 11.5 | 34.6 | 83.7 | 24.3 | 26.8 | 13.5 | 26 | Hb E-trait | E |
| 44 | M | 4.41 | 11.7 | 35 | 79.3 | 26.5 | 33.2 | 12.8 | 30.9 | Hb E-trait | E |
| 35 | M | 5.23 | 12.7 | 38.2 | 73.1 | 23.4 | 28.3 | 13.6 | 29.4 | Hb E-trait | E |
| 23 | M | 3.93 | 9.9 | 29.7 | 75.6 | 24.6 | 32.5 | 14.5 | 29.2 | Hb E-trait | E |
| 26 | M | 4.29 | 11.8 | 35.4 | 82.5 | 24.6 | 32.4 | 13.6 | 26.2 | Hb E-trait | E |
| 28 | M | 4.30 | 12.0 | 36 | 83.8 | 27.9 | 33.2 | 12.6 | 29.4 | Hb E-trait | E |
| 38 | M | 4.44 | 11.7 | 35 | 78.8 | 23.6 | 28.4 | 14.1 | 29 | Hb E-trait | E |
| 19 | M | 4.83 | 11.7 | 35 | 72.5 | 21.9 | 22.6 | 12.8 | 28.5 | Hb E-trait | E |
| 26 | M | 4.94 | 12.0 | 36 | 72.9 | 24.3 | 26.3 | 13.6 | 27.8 | Hb E-trait | E |
| 23 | M | 4.87 | 12.0 | 36.1 | 74.1 | 24.6 | 33.2 | 13.5 | 29.9 | Hb E-trait | E |
| 26 | M | 4.61 | 11.7 | 35.2 | 76.4 | 24.3 | 27.2 | 13.5 | 22.2 | Hb E-trait | E |
| 31 | M | 4.87 | 12.7 | 38 | 78 | 23.8 | 26.8 | 12.6 | 26.5 | Hb E-trait | E |
| 30 | M | 4.32 | 12.3 | 36.8 | 85.1 | 24.8 | 26.3 | 12.8 | 26.2 | Hb E-trait | E |
| 36 | M | 4.63 | 12.0 | 36.1 | 78 | 22.4 | 24.3 | 13.5 | 26.7 | Hb E-trait | E |
| 31 | M | 4.89 | 13.0 | 39 | 79.8 | 25.3 | 28.4 | 14.2 | 30.3 | Hb E-trait | E |
| 19 | M | 4.53 | 11.9 | 35.8 | 79 | 26.6 | 25.4 | 12.4 | 31.6 | Hb E-trait | E |
| 28 | M | 4.80 | 12.0 | 36 | 75 | 25.3 | 28.1 | 12.8 | 25.3 | Hb E-trait | SEA/E |
| 26 | M | 4.80 | 9.0 | 27 | 51 | 19 | 26.1 | 14.5 | 16.3 | Hb E-trait | SEA/E |
| 28 | M | 4.43 | 11.7 | 35 | 79 | 20.9 | 21.8 | 12.4 | 29.2 | Hb E-trait | E |
| 25 | M | 4.50 | 12.0 | 36 | 80 | 26.7 | 33.2 | 12.6 | 29.8 | Hb E-trait | E |
| 27 | M | 5.83 | 10.7 | 32 | 54.9 | 24.3 | 26.8 | 12.8 | 15.3 | Hb E-trait | SEA/E |
| 36 | M | 4.21 | 11.5 | 34.5 | 82 | 24.5 | 26.4 | 12.8 | 31.8 | Hb E-trait | E |
| 33 | M | 4.13 | 11.5 | 34.6 | 83.7 | 24.3 | 26.8 | 13.5 | 26 | Hb E-trait | E |
| 45 | M | 4.41 | 11.7 | 35 | 79.3 | 26.5 | 33.2 | 12.8 | 30.9 | Hb E-trait | E |
| 35 | M | 5.23 | 12.7 | 38.2 | 73.1 | 23.4 | 28.3 | 13.6 | 29.4 | Hb E-trait | E |
| 29 | M | 3.96 | 9.9 | 29.7 | 75.6 | 24.6 | 32.5 | 14.3 | 25.4 | Hb E-trait | E |
| 26 | M | 4.29 | 11.8 | 35.4 | 82.5 | 24.6 | 32.4 | 13.6 | 26.2 | Hb E-trait | E |
| 28 | M | 4.30 | 12.0 | 36 | 83.8 | 27.9 | 33.2 | 12.6 | 29.4 | Hb E-trait | E |
| 30 | M | 4.68 | 11.0 | 33 | 70.5 | 23.2 | 26.4 | 13.5 | 26.2 | Hb E-trait | E |
| 19 | M | 4.83 | 11.7 | 35 | 72.5 | 21.9 | 22.6 | 12.8 | 28.5 | Hb E-trait | E |
| 26 | M | 4.94 | 12.0 | 36 | 72.9 | 24.3 | 26.3 | 13.6 | 27.8 | Hb E-trait | E |
| 30 | M | 5.56 | 11.7 | 35 | 62.9 | 23.4 | 27.3 | 13.2 | 21.4 | Hb E-trait | SEA/E |
| 23 | M | 4.87 | 12.0 | 36.1 | 74.1 | 24.6 | 33.2 | 13.5 | 29.9 | Hb E-trait | E |
| 25 | M | 4.61 | 11.7 | 35.2 | 76.4 | 24.3 | 27.2 | 13.5 | 22.2 | Hb E-trait | E |
| 29 | M | 4.27 | 11.7 | 35.2 | 82.4 | 25.3 | 28.3 | 14.2 | 26.4 | Hb E-trait | E |
| 31 | M | 4.87 | 12.7 | 38 | 78 | 23.8 | 26.8 | 12.6 | 26.5 | Hb E-trait | E |
| 23 | M | 4.87 | 12.0 | 36.1 | 74.1 | 24.6 | 33.2 | 13.5 | 29.9 | Hb E-trait | E |
| 25 | M | 4.61 | 11.7 | 35.2 | 76.4 | 24.3 | 27.2 | 13.5 | 22.2 | Hb E-trait | E |
| 31 | M | 4.87 | 12.7 | 38 | 78 | 23.8 | 26.8 | 12.6 | 26.5 | Hb E-trait | E |
| 30 | M | 4.32 | 12.3 | 36.8 | 85.1 | 24.8 | 26.3 | 12.8 | 26.2 | Hb E-trait | E |
| 36 | M | 4.63 | 12.0 | 36.1 | 78 | 22.4 | 24.3 | 13.5 | 26.7 | Hb E-trait | E |
| 31 | M | 4.89 | 13.0 | 39 | 79.8 | 25.3 | 28.4 | 14.2 | 30.3 | Hb E-trait | E |
| 19 | M | 4.53 | 11.9 | 35.8 | 79 | 26.6 | 25.4 | 12.4 | 31.6 | Hb E-trait | E |
| 37 | M | 4.51 | 11.7 | 35 | 77.6 | 23.5 | 26.4 | 13.6 | 29 | Hb E-trait | E |
| 24 | M | 4.68 | 11.0 | 33 | 70.5 | 23.2 | 26.4 | 13.5 | 15.8 | Hb E-trait | SEA/E |
| 30 | M | 5.37 | 12.0 | 36 | 67 | 25.3 | 28.4 | 14.1 | 19 | Hb E-trait | SEA/E |
| 27 | M | 4.46 | 11.7 | 35 | 78.5 | 23.2 | 25.4 | 12.8 | 26.3 | Hb E-trait | E |
| 32 | M | 4.71 | 12.0 | 36 | 76.4 | 24.3 | 27.2 | 13.5 | 28.7 | Hb E-trait | E |
| 36 | M | 5.48 | 12.7 | 38 | 69.4 | 23.8 | 26.8 | 12.6 | 20.1 | Hb E-trait | SEA/E |
| 22 | M | 4.86 | 12.7 | 38 | 78.2 | 24.8 | 26.3 | 12.8 | 32.9 | Hb E-trait | E |
| 29 | M | 4.51 | 11.7 | 35 | 77.6 | 23.5 | 26.4 | 13.6 | 29 | Hb E-trait | E |
| 24 | M | 4.68 | 11.0 | 33 | 70.5 | 23.2 | 26.4 | 13.5 | 15.8 | Hb E-trait | SEA/E |
| 29 | M | 4.68 | 12.0 | 36 | 67 | 25.3 | 28.4 | 14.1 | 19 | Hb E-trait | SEA/E |
| 35 | M | 4.46 | 11.7 | 35 | 78.5 | 23.2 | 25.4 | 12.8 | 26.3 | Hb E-trait | E |
| 26 | M | 4.94 | 12.0 | 36 | 72.9 | 24.3 | 26.3 | 13.6 | 27.8 | Hb E-trait | E |
| 23 | M | 4.87 | 12.0 | 36.1 | 74.1 | 24.6 | 33.2 | 13.5 | 29.9 | Hb E-trait | E |
| 31 | M | 4.61 | 11.7 | 35.2 | 76.4 | 24.3 | 27.2 | 13.5 | 22.2 | Hb E-trait | E |
| 39 | M | 4.27 | 11.7 | 35.2 | 82.4 | 25.3 | 28.3 | 14.2 | 26.4 | Hb E-trait | E |
| 31 | M | 4.87 | 12.7 | 38 | 78 | 23.8 | 26.8 | 12.6 | 26.5 | Hb E-trait | E |
| 36 | M | 4.21 | 11.5 | 34.5 | 82 | 24.5 | 26.4 | 12.8 | 31.8 | Hb E-trait | E |
| 36 | M | 4.41 | 11.7 | 35 | 79.3 | 26.5 | 33.2 | 12.8 | 30.9 | Hb E-trait | E |
| 37 | M | 3.93 | 9.9 | 29.7 | 75.6 | 24.6 | 32.5 | 14.5 | 29.2 | Hb E-trait | E |
| 26 | M | 4.29 | 11.8 | 35.4 | 82.5 | 24.6 | 32.4 | 13.6 | 26.2 | Hb E-trait | E |
| 28 | M | 4.30 | 12.0 | 36 | 83.8 | 27.9 | 33.2 | 12.6 | 29.4 | Hb E-trait | E |
| 24 | M | 4.49 | 11.7 | 35 | 78 | 23.2 | 26.4 | 13.6 | 28.2 | Hb E-trait | E |
| 19 | M | 4.83 | 11.7 | 35 | 72.5 | 21.9 | 22.6 | 12.8 | 28.5 | Hb E-trait | E |
| 26 | M | 4.94 | 12.0 | 36 | 72.9 | 24.3 | 26.3 | 13.6 | 27.8 | Hb E-trait | E |
| 23 | M | 4.87 | 12.0 | 36.1 | 74.1 | 24.6 | 33.2 | 13.5 | 29.9 | Hb E-trait | E |
| 25 | M | 4.61 | 11.7 | 35.2 | 76.4 | 24.3 | 27.2 | 13.5 | 22.2 | Hb E-trait | E |
| 35 | M | 4.54 | 11.7 | 35 | 77.1 | 24.5 | 26.4 | 12.8 | 31.7 | Hb E-trait | E |
| 32 | M | 4.18 | 11.0 | 33 | 79 | 25.4 | 27.4 | 14.3 | 29.6 | Hb E-trait | E |
| 28 | M | 4.25 | 11.3 | 34 | 80 | 24.5 | 26.3 | 13.5 | 27.6 | Hb E-trait | E |
| 26 | M | 4.93 | 11.7 | 35 | 71 | 23 | 25 | 12.6 | 13.9 | Hb E-trait | SEA/E |
| 33 | M | 5.00 | 13.3 | 40 | 80 | 25.4 | 27.5 | 13.5 | 27 | Hb E-trait | E |
| 28 | M | 4.95 | 14.1 | 42.2 | 85.3 | 23.4 | 25.6 | 13.5 | 26.6 | Hb E-trait | E |
| 30 | M | 5.85 | 12.7 | 38 | 65 | 13.8 | 18.3 | 13.6 | 20.9 | Hb E-trait | SEA/E |
| 28 | M | 5.51 | 12.7 | 38 | 69 | 23 | 25.9 | 13.5 | 19.3 | Hb E-trait | SEA/E |
| 30 | M | 4.82 | 13.3 | 40 | 83 | 23.6 | 26.5 | 14.1 | 26.2 | Hb E-trait | E |
| 40 | M | 4.35 | 10.7 | 32 | 73.6 | 24.5 | 26.9 | 12.6 | 28.9 | Hb E-trait | E |
| 35 | M | 4.45 | 12.7 | 38 | 85 | 28.5 | 33.2 | 13.5 | 26.9 | Hb E-trait | E |
| 36 | M | 5.13 | 13.4 | 40.2 | 78.3 | 24.6 | 26.3 | 13.6 | 26.7 | Hb E-trait | E |
| 22 | M | 4.71 | 13.0 | 39 | 82.8 | 25.3 | 28.4 | 14.2 | 27.9 | Hb E-trait | E |
| 36 | M | 4.53 | 12.0 | 36 | 79.5 | 23.2 | 25.4 | 12.4 | 24.8 | Hb E-trait | E |
| 30 | M | 5.85 | 12.7 | 38 | 65 | 13.8 | 18.3 | 13.6 | 20.9 | Hb E-trait | SEA/E |
| 35 | M | 4.82 | 13.3 | 40 | 83 | 23.6 | 26.5 | 14.1 | 26.2 | Hb E-trait | E |
| 26 | M | 4.35 | 10.7 | 32 | 73.6 | 24.5 | 26.9 | 12.6 | 28.9 | Hb E-trait | E |
| 31 | M | 4.26 | 11.0 | 33 | 77.5 | 20.1 | 22.6 | 14.2 | 25.9 | Hb E-trait | E |
| 35 | M | 5.20 | 12.7 | 38 | 73.1 | 25.4 | 28.6 | 13.5 | 26.9 | Hb E-trait | E |
| 25 | M | 4.12 | 11.7 | 35 | 85 | 25.4 | 27.3 | 13.5 | 29.6 | Hb E-trait | E |
| 26 | M | 4.35 | 10.7 | 32 | 73.6 | 24.5 | 26.9 | 12.6 | 28.9 | Hb E-trait | E |
| 31 | M | 4.26 | 11.0 | 33 | 77.5 | 20.1 | 22.6 | 14.2 | 25.9 | Hb E-trait | E |
| 36 | M | 5.13 | 13.4 | 40.2 | 78.3 | 24.6 | 26.3 | 13.6 | 26.7 | Hb E-trait | E |
| 36 | M | 4.24 | 11.2 | 33.7 | 79.5 | 23.2 | 25.4 | 12.4 | 24.8 | Hb E-trait | E |
| 33 | M | 4.21 | 11.5 | 34.5 | 82 | 24.5 | 26.4 | 12.8 | 31.8 | Hb E-trait | E |
| 35 | M | 5.23 | 12.7 | 38.2 | 73.1 | 23.4 | 28.3 | 13.6 | 29.4 | Hb E-trait | E |
| 26 | M | 4.29 | 11.8 | 35.4 | 82.5 | 24.6 | 32.4 | 13.6 | 26.2 | Hb E-trait | E |
| 30 | M | 5.85 | 12.7 | 38 | 65 | 13.8 | 18.3 | 13.6 | 20.9 | Hb E-trait | SEA/E |
| 35 | M | 4.82 | 13.3 | 40 | 83 | 23.6 | 26.5 | 14.1 | 26.2 | Hb E-trait | E |
| 26 | M | 4.35 | 10.7 | 32 | 73.6 | 24.5 | 26.9 | 12.6 | 28.9 | Hb E-trait | E |
| 31 | M | 4.18 | 11.0 | 33 | 78.9 | 20.1 | 22.6 | 14.2 | 25.9 | Hb E-trait | E |
| 36 | M | 4.63 | 12.0 | 36.1 | 78 | 22.4 | 24.3 | 13.5 | 26.7 | Hb E-trait | E |
| 31 | M | 4.89 | 13.0 | 39 | 79.8 | 25.3 | 28.4 | 14.2 | 30.3 | Hb E-trait | E |
| 19 | M | 4.48 | 11.9 | 35.8 | 80 | 26.6 | 25.4 | 12.4 | 31.6 | Hb E-trait | E |
| 28 | M | 4.14 | 12.0 | 36 | 87 | 25.3 | 28.1 | 12.8 | 25.3 | Hb E-trait | E |
| 23 | M | 4.87 | 12.0 | 36.1 | 74.1 | 24.6 | 33.2 | 13.5 | 29.9 | Hb E-trait | E |
| 36 | M | 4.61 | 11.7 | 35.2 | 76.4 | 24.3 | 27.2 | 13.5 | 22.2 | Hb E-trait | E |
| 29 | M | 4.27 | 11.7 | 35.2 | 82.4 | 25.3 | 28.3 | 14.2 | 26.4 | Hb E-trait | E |
| 31 | M | 4.87 | 12.7 | 38 | 78 | 23.8 | 26.8 | 12.6 | 26.5 | Hb E-trait | E |
| 36 | M | 4.21 | 11.5 | 34.5 | 82 | 24.5 | 26.4 | 12.8 | 31.8 | Hb E-trait | E |
| 36 | M | 5.68 | 11.3 | 34 | 59.9 | 21.2 | 23.1 | 13.5 | 19.3 | Hb E-trait | SEA/E |
| 19 | M | 4.83 | 11.7 | 35 | 72.5 | 21.9 | 22.6 | 12.8 | 28.5 | Hb E-trait | E |
| 26 | M | 4.69 | 11.4 | 34.2 | 72.9 | 24.3 | 26.3 | 13.6 | 27.8 | Hb E-trait | E |
| 23 | M | 4.87 | 12.0 | 36.1 | 74.1 | 24.6 | 33.2 | 13.5 | 29.9 | Hb E-trait | E |
| 25 | M | 4.61 | 11.7 | 35.2 | 76.4 | 24.3 | 27.2 | 13.5 | 22.2 | Hb E-trait | E |
| 35 | M | 4.54 | 11.7 | 35 | 77.1 | 24.5 | 26.4 | 12.8 | 31.7 | Hb E-trait | E |
| 32 | M | 4.18 | 11.0 | 33 | 79 | 25.4 | 27.4 | 14.3 | 29.6 | Hb E-trait | E |
| 23 | M | 4.87 | 12.0 | 36.1 | 74.1 | 24.6 | 33.2 | 13.5 | 29.9 | Hb E-trait | E |
| 31 | M | 4.61 | 11.7 | 35.2 | 76.4 | 24.3 | 27.2 | 13.5 | 22.2 | Hb E-trait | E |
| 39 | M | 4.27 | 11.7 | 35.2 | 82.4 | 25.3 | 28.3 | 14.2 | 26.4 | Hb E-trait | E |
| 32 | M | 4.18 | 11.0 | 33 | 79 | 25.4 | 27.4 | 14.3 | 29.6 | Hb E-trait | E |
| 28 | M | 4.43 | 11.7 | 35 | 79 | 20.9 | 21.8 | 12.4 | 29.2 | Hb E-trait | E |
| 25 | M | 4.50 | 12.0 | 36 | 80 | 26.7 | 33.2 | 12.6 | 29.8 | Hb E-trait | E |
| 30 | M | 5.83 | 10.7 | 32 | 54.9 | 24.3 | 26.8 | 12.8 | 19.5 | Hb E-trait | SEA/E |
| 36 | M | 4.21 | 11.5 | 34.5 | 82 | 24.5 | 26.4 | 12.8 | 31.8 | Hb E-trait | E |
| 33 | M | 4.13 | 11.5 | 34.6 | 83.7 | 24.3 | 26.8 | 13.5 | 26 | Hb E-trait | E |
| 35 | M | 3.91 | 9.9 | 29.7 | 76 | 24.6 | 32.5 | 14.4 | 29.0 | Hb E-trait | E |
| 26 | M | 4.44 | 11.8 | 35.4 | 79.8 | 24.6 | 32.4 | 13.6 | 26.2 | Hb E-trait | E |
| 28 | M | 4.50 | 12.0 | 36 | 80 | 26.7 | 33.2 | 12.6 | 29.4 | Hb E-trait | E |
| 24 | M | 4.49 | 11.7 | 35 | 78 | 23.2 | 26.4 | 13.6 | 28.2 | Hb E-trait | E |
| 33 | M | 4.21 | 11.5 | 34.5 | 82 | 24.5 | 26.4 | 12.8 | 31.8 | Hb E-trait | E |
| 33 | M | 4.13 | 11.5 | 34.6 | 83.7 | 24.3 | 26.8 | 13.5 | 26 | Hb E-trait | E |
| 35 | M | 5.23 | 12.7 | 38.2 | 73.1 | 23.4 | 28.3 | 13.6 | 29.4 | Hb E-trait | E |
| 32 | M | 4.01 | 9.9 | 29.7 | 75.6 | 24.6 | 32.5 | 14.5 | 29.2 | Hb E-trait | E |
| 26 | M | 4.29 | 11.8 | 35.4 | 82.5 | 24.6 | 32.4 | 13.6 | 26.2 | Hb E-trait | E |
| 28 | M | 4.30 | 12.0 | 36 | 83.8 | 27.9 | 33.2 | 12.6 | 29.4 | Hb E-trait | E |
| 38 | M | 4.44 | 11.7 | 35 | 78.8 | 23.6 | 28.4 | 14.1 | 29 | Hb E-trait | E |
| 19 | M | 4.83 | 11.7 | 35 | 72.5 | 21.9 | 22.6 | 12.8 | 28.5 | Hb E-trait | E |
| 26 | M | 4.94 | 12.0 | 36 | 72.9 | 24.3 | 26.3 | 12.6 | 27.8 | Hb E-trait | E |

| **Aged** | **Gender** | **RBC**  **(million/uL)** | **Hb**  **(g/dL)** | **Hct**  **(%)** | **MCV**  **(fL)** | **MCH**  **(pg)** | **MCHC**  **(g/dL)** | **RDW**  **(%)** | **HbE level (%)** | **Hemoglobin typing** | **Grouping** |
| --- | --- | --- | --- | --- | --- | --- | --- | --- | --- | --- | --- |
| 38 | F | 4.87 | 12.0 | 36 | 73.9 | 27.8 | 29.8 | 12.8 | 28.3 | Hb E-trait | E |
| 24 | F | 4.77 | 10.7 | 32 | 67.1 | 23 | 26 | 13.6 | 21.9 | Hb E-trait | E |
| 27 | F | 4.19 | 11.0 | 33 | 78.8 | 23 | 25.8 | 14.2 | 25.9 | Hb E-trait | E |
| 38 | F | 4.63 | 12.7 | 38 | 82 | 25.3 | 28.1 | 13.2 | 27.6 | Hb E-trait | E |
| 23 | F | 5.31 | 12.7 | 38.2 | 72 | 25.6 | 28.3 | 13.6 | 27.4 | Hb E-trait | E |
| 25 | F | 5.34 | 12.0 | 36.1 | 67.6 | 22.4 | 24.5 | 14.2 | 20.9 | Hb E-trait | SEA/E |
| 43 | F | 4.27 | 11.7 | 35 | 82 | 25.3 | 28.4 | 12.6 | 27.1 | Hb E-trait | E |
| 22 | F | 3.98 | 11.7 | 35 | 88 | 24.9 | 26.3 | 12.8 | 29.7 | Hb E-trait | E |
| 28 | F | 4.81 | 12.3 | 37 | 77 | 25.3 | 27.8 | 13.2 | 27.5 | Hb E-trait | E |
| 30 | F | 4.43 | 11.7 | 35 | 79 | 25.4 | 27.3 | 13.5 | 29.6 | Hb E-trait | E |
| 33 | F | 4.93 | 12.0 | 36 | 73 | 24.3 | 26.8 | 13.5 | 27 | Hb E-trait | E |
| 35 | F | 4.02 | 11.0 | 33 | 82 | 23.6 | 26.2 | 12.8 | 27 | Hb E-trait | E |
| 23 | F | 5.67 | 11.7 | 35 | 61.7 | 20.6 | 26.3 | 13.6 | 19.7 | Hb E-trait | SEA/E |
| 31 | F | 4.26 | 11.0 | 33 | 77.5 | 20.1 | 22.6 | 14.2 | 25.9 | Hb E-trait | E |
| 33 | F | 4.93 | 12.0 | 36 | 73 | 20.3 | 23.1 | 13.2 | 21.8 | Hb E-trait | E |
| 23 | F | 4.50 | 12.0 | 36.1 | 80.2 | 25.4 | 27.3 | 12.6 | 29 | Hb E-trait | E |
| 20 | F | 4.67 | 11.7 | 35 | 75 | 24.3 | 26.8 | 13.6 | 28.3 | Hb E-trait | E |
| 35 | F | 5.21 | 12.7 | 38 | 72.9 | 24.4 | 27.4 | 14.1 | 30.1 | Hb E-trait | E |
| 27 | F | 4.07 | 10.7 | 32 | 78.6 | 24.3 | 26.8 | 12.8 | 26 | Hb E-trait | E |
| 26 | F | 4.14 | 11.0 | 33 | 79.7 | 25.5 | 27.3 | 13.2 | 29.2 | Hb E-trait | E |
| 26 | F | 4.36 | 11.3 | 34 | 78 | 24.3 | 26.8 | 13.5 | 26 | Hb E-trait | E |
| 27 | F | 4.67 | 12.0 | 36 | 77.1 | 24.3 | 26.3 | 13.6 | 25.7 | Hb E-trait | E |
| 31 | F | 4.38 | 11.1 | 33.3 | 76 | 25.5 | 28.2 | 14.2 | 29.1 | Hb E-trait | E |
| 38 | F | 4.71 | 12.0 | 36 | 76.4 | 24.3 | 27.2 | 13.5 | 28.7 | Hb E-trait | E |
| 24 | F | 4.55 | 11.7 | 35 | 76.9 | 24.2 | 26.4 | 13.6 | 25 | Hb E-trait | E |
| 31 | F | 4.43 | 11.7 | 35 | 79 | 25.3 | 28.3 | 14.2 | 29.5 | Hb E-trait | E |
| 26 | F | 4.61 | 12.3 | 37 | 80.3 | 23 | 26.1 | 12.4 | 31.3 | Hb E-trait | E |
| 22 | F | 4.50 | 13.3 | 40 | 89 | 29.5 | 33.2 | 13.2 | 26 | Hb E-trait | E |
| 38 | F | 4.10 | 11.7 | 35 | 85.4 | 24.5 | 26.8 | 13.6 | 31.3 | Hb E-trait | E |
| 27 | F | 4.53 | 11.3 | 34 | 75 | 22.4 | 24.3 | 13.5 | 19.1 | Hb E-trait | SEA/E |
| 30 | F | 4.21 | 10.7 | 32 | 76 | 25.3 | 28.4 | 14.1 | 28.2 | Hb E-trait | SEA/E |
| 35 | F | 4.17 | 11.0 | 33 | 79.2 | 25.5 | 28.3 | 13.5 | 29.1 | Hb E-trait | E |
| 26 | F | 4.53 | 11.0 | 33 | 72.8 | 25.5 | 27.3 | 13.2 | 29.2 | Hb E-trait | E |
| 24 | F | 4.49 | 11.7 | 35 | 78 | 23.2 | 26.4 | 13.6 | 28.2 | Hb E-trait | E |
| 30 | F | 4.68 | 11.0 | 33 | 70.5 | 23.2 | 26.4 | 13.5 | 26.2 | Hb E-trait | E |
| 38 | F | 4.44 | 11.7 | 35 | 78.8 | 23.6 | 28.4 | 14.1 | 29 | Hb E-trait | E |
| 31 | F | 5.68 | 11.3 | 34 | 59.9 | 19.9 | 23.1 | 13.5 | 14.3 | Hb E-trait | SEA/E |
| 23 | F | 4.47 | 11.3 | 34 | 76 | 25.3 | 33.2 | 12.6 | 26 | Hb E-trait | E |
| 19 | F | 4.38 | 11.1 | 33.3 | 76 | 25.5 | 28.2 | 14.2 | 29.1 | Hb E-trait | E |
| 28 | F | 4.74 | 12.0 | 36 | 76 | 23.6 | 26.4 | 13.6 | 27.8 | Hb E-trait | E |
| 23 | F | 4.27 | 11.7 | 35.2 | 82.4 | 25.3 | 28.3 | 14.2 | 26.4 | Hb E-trait | E |
| 26 | F | 4.61 | 12.3 | 37 | 80.3 | 23 | 26.1 | 12.4 | 29.5 | Hb E-trait | E |
| 22 | F | 4.50 | 13.3 | 40 | 89 | 29.5 | 33.2 | 13.2 | 26 | Hb E-trait | E |
| 24 | F | 5.38 | 11.7 | 35 | 65 | 24.5 | 26.8 | 13.6 | 19.6 | Hb E-trait | SEA/E |
| 32 | F | 4.21 | 10.7 | 32 | 76 | 25.3 | 28.4 | 14.1 | 31.7 | Hb E-trait | E |
| 30 | F | 4.38 | 11.0 | 33 | 75.4 | 25.5 | 28.3 | 13.5 | 26 | Hb E-trait | E |
| 24 | F | 5.13 | 13.4 | 40.2 | 78.3 | 26 | 26.3 | 13.6 | 28.8 | Hb E-trait | E |
| 29 | F | 4.75 | 12.7 | 38 | 80 | 24 | 26.4 | 13.2 | 30.1 | Hb E-trait | E |
| 28 | F | 4.50 | 11.7 | 35 | 77.7 | 24.9 | 26.8 | 13.6 | 31.1 | Hb E-trait | E |
| 24 | F | 5.03 | 12.3 | 37 | 73.5 | 24.5 | 33.2 | 13.5 | 27.5 | Hb E-trait | E |
| 21 | F | 5.67 | 12.7 | 38 | 67 | 25.9 | 27.4 | 14.1 | 20.7 | Hb E-trait | SEA/E |
| 34 | F | 4.07 | 10.7 | 32 | 78.6 | 24.3 | 26.8 | 12.8 | 30.2 | Hb E-trait | E |
| 26 | F | 4.53 | 11.0 | 33 | 72.8 | 25.5 | 27.3 | 13.2 | 29.2 | Hb E-trait | E |
| 24 | F | 4.49 | 11.7 | 35 | 78 | 23.2 | 26.4 | 13.6 | 28.2 | Hb E-trait | E |
| 38 | F | 4.44 | 11.7 | 35 | 78.8 | 23.6 | 28.4 | 14.1 | 29 | Hb E-trait | E |
| 31 | F | 5.68 | 11.3 | 34 | 59.9 | 21.2 | 23.1 | 13.5 | 14.3 | Hb E-trait | SEA/E |
| 23 | F | 4.47 | 11.3 | 34 | 76 | 25.3 | 33.2 | 12.6 | 26 | Hb E-trait | E |
| 19 | F | 4.38 | 11.1 | 33.3 | 76 | 25.5 | 28.2 | 14.2 | 29.1 | Hb E-trait | E |
| 28 | F | 4.74 | 12.0 | 36 | 76 | 23.6 | 26.4 | 13.6 | 27.8 | Hb E-trait | E |
| 26 | F | 4.61 | 12.3 | 37 | 80.3 | 23 | 26.1 | 12.4 | 29.5 | Hb E-trait | E |
| 26 | F | 4.53 | 11.0 | 33 | 72.8 | 25.5 | 27.3 | 13.2 | 29.2 | Hb E-trait | E |
| 24 | F | 4.49 | 11.7 | 35 | 78 | 23.2 | 26.4 | 13.6 | 28.2 | Hb E-trait | E |
| 25 | F | 4.68 | 11.0 | 33 | 70.5 | 23.2 | 26.4 | 13.5 | 26.2 | Hb E-trait | E |
| 31 | F | 5.68 | 11.3 | 34 | 59.9 | 21.2 | 23.1 | 13.5 | 14.3 | Hb E-trait | SEA/E |
| 23 | F | 4.47 | 11.3 | 34 | 76 | 25.3 | 33.2 | 12.6 | 26 | Hb E-trait | E |
| 19 | F | 4.38 | 11.1 | 33.3 | 76 | 25.5 | 28.2 | 14.2 | 29.1 | Hb E-trait | E |
| 28 | F | 4.74 | 12.0 | 36 | 76 | 23.6 | 26.4 | 13.6 | 27.8 | Hb E-trait | E |
| 23 | F | 4.27 | 11.7 | 35.2 | 82.4 | 25.3 | 28.3 | 14.2 | 26.4 | Hb E-trait | E |
| 26 | F | 4.61 | 12.3 | 37 | 80.3 | 23 | 26.1 | 12.4 | 29.5 | Hb E-trait | E |
| 22 | F | 4.50 | 13.3 | 40 | 89 | 29.6 | 33.2 | 13.2 | 26 | Hb E-trait | E |
| 24 | F | 5.38 | 11.7 | 35 | 65 | 24.5 | 26.8 | 13.6 | 19.6 | Hb E-trait | SEA/E |
| 32 | F | 4.21 | 10.7 | 32 | 76 | 25.3 | 28.4 | 14.1 | 31.7 | Hb E-trait | E |
| 30 | F | 4.38 | 11.0 | 33 | 75.4 | 25.5 | 28.3 | 13.5 | 26 | Hb E-trait | E |
| 24 | F | 5.13 | 13.4 | 40.2 | 78.3 | 26 | 26.3 | 13.6 | 26.4 | Hb E-trait | E |
| 29 | F | 4.75 | 12.7 | 38 | 80 | 24 | 26.4 | 13.2 | 30.1 | Hb E-trait | E |
| 28 | F | 4.50 | 11.7 | 35 | 77.7 | 24.9 | 26.8 | 13.6 | 31.1 | Hb E-trait | E |
| 24 | F | 5.03 | 12.3 | 37 | 73.5 | 24.5 | 33.2 | 13.5 | 27.5 | Hb E-trait | E |
| 18 | F | 5.67 | 12.7 | 38 | 67 | 25.9 | 27.4 | 14.1 | 20.7 | Hb E-trait | SEA/E |
| 34 | F | 4.07 | 10.7 | 32 | 78.6 | 24.3 | 26.8 | 13 | 30.2 | Hb E-trait | E |
| 26 | F | 4.53 | 11.0 | 33 | 72.8 | 25.5 | 27.3 | 13.2 | 29.2 | Hb E-trait | E |
| 24 | F | 4.49 | 11.7 | 35 | 78 | 23.2 | 26.4 | 13.6 | 28.2 | Hb E-trait | E |
| 38 | F | 4.44 | 11.7 | 35 | 78.8 | 23.6 | 28.4 | 14.1 | 29 | Hb E-trait | E |
| 31 | F | 5.68 | 11.3 | 34 | 59.9 | 21.2 | 23.1 | 13.5 | 14.3 | Hb E-trait | SEA/E |
| 26 | F | 4.47 | 11.3 | 34 | 76 | 25.3 | 33.2 | 12.6 | 26 | Hb E-trait | E |
| 19 | F | 4.38 | 11.1 | 33.3 | 76 | 25.5 | 28.2 | 14.2 | 29.1 | Hb E-trait | E |
| 28 | F | 4.74 | 12.0 | 36 | 76 | 23.6 | 26.4 | 13.6 | 27.8 | Hb E-trait | E |
| 26 | F | 4.61 | 12.3 | 37 | 80.3 | 23 | 26.1 | 12.4 | 29.5 | Hb E-trait | E |
| 36 | F | 4.32 | 12.3 | 36.8 | 85.1 | 24.8 | 26.3 | 12.8 | 26.2 | Hb E-trait | E |
| 28 | F | 4.74 | 12.0 | 36 | 76 | 23.6 | 26.4 | 13.6 | 27.8 | Hb E-trait | E |
| 23 | F | 4.27 | 11.7 | 35.2 | 82.4 | 25.3 | 28.3 | 14.2 | 26.4 | Hb E-trait | E |
| 26 | F | 4.61 | 12.3 | 37 | 80.3 | 23 | 26.1 | 12.4 | 29.5 | Hb E-trait | E |
| 22 | F | 4.50 | 13.3 | 40 | 89 | 29.6 | 33.2 | 13.2 | 26 | Hb E-trait | E |
| 24 | F | 5.38 | 11.7 | 35 | 65 | 24.5 | 26.8 | 13.6 | 19.6 | Hb E-trait | SEA/E |
| 32 | F | 4.21 | 10.7 | 32 | 76 | 25.3 | 28.4 | 14.1 | 31.7 | Hb E-trait | E |
| 30 | F | 4.38 | 11.0 | 33 | 75.4 | 25.5 | 28.3 | 13.5 | 26 | Hb E-trait | E |
| 24 | F | 5.13 | 13.4 | 40.2 | 78.3 | 26 | 26.3 | 13.6 | 28.8 | Hb E-trait | E |
| 26 | F | 4.14 | 11.0 | 33 | 79.7 | 25.5 | 27.3 | 13.2 | 29.2 | Hb E-trait | E |
| 26 | F | 4.36 | 11.3 | 34 | 78 | 24.3 | 26.8 | 13.5 | 26 | Hb E-trait | E |
| 27 | F | 4.67 | 12.0 | 36 | 77.1 | 24.3 | 26.3 | 13.6 | 25.7 | Hb E-trait | E |
| 31 | F | 4.38 | 11.1 | 33.3 | 76 | 25.5 | 28.2 | 14.2 | 29.1 | Hb E-trait | E |
| 24 | F | 4.55 | 11.7 | 35 | 76.9 | 24.2 | 26.4 | 13.6 | 25 | Hb E-trait | E |
| 31 | F | 4.43 | 11.7 | 35 | 79 | 25.3 | 28.3 | 14.2 | 29.5 | Hb E-trait | E |
| 26 | F | 4.61 | 12.3 | 37 | 80.3 | 23 | 26.1 | 12.4 | 31.3 | Hb E-trait | E |
| 22 | F | 4.50 | 13.3 | 40 | 89 | 29.6 | 33.2 | 13.2 | 26 | Hb E-trait | E |
| 38 | F | 4.10 | 11.7 | 35 | 85.4 | 24.5 | 26.8 | 13.6 | 31.3 | Hb E-trait | E |
| 27 | F | 4.53 | 11.3 | 34 | 75 | 22.4 | 24.3 | 13.5 | 19.1 | Hb E-trait | SEA/E |
| 30 | F | 4.21 | 10.7 | 32 | 76 | 25.3 | 28.4 | 14.1 | 28.2 | Hb E-trait | SEA/E |
| 25 | F | 4.14 | 11.0 | 33 | 79.7 | 25.5 | 27.3 | 13.2 | 29.2 | Hb E-trait | E |
| 26 | F | 4.36 | 11.3 | 34 | 78 | 24.3 | 26.8 | 13.5 | 26 | Hb E-trait | E |
| 32 | F | 4.67 | 12.0 | 36 | 77.1 | 24.3 | 26.3 | 13.6 | 25.7 | Hb E-trait | E |
| 31 | F | 4.38 | 11.1 | 33.3 | 76 | 25.5 | 28.2 | 14.2 | 29.1 | Hb E-trait | E |
| 19 | F | 4.83 | 11.7 | 35 | 72.5 | 21.9 | 22.6 | 12.8 | 28.5 | Hb E-trait | E |
| 19 | F | 4.38 | 11.1 | 33.3 | 76 | 25.5 | 28.2 | 14.2 | 29.1 | Hb E-trait | E |
| 28 | F | 4.74 | 12.0 | 36 | 76 | 23.6 | 26.4 | 13.6 | 27.8 | Hb E-trait | E |
| 38 | F | 4.61 | 12.3 | 37 | 80.3 | 23 | 26.1 | 12.4 | 29.5 | Hb E-trait | E |
| 36 | F | 4.32 | 12.3 | 36.8 | 85.1 | 24.8 | 26.3 | 12.8 | 26.2 | Hb E-trait | E |
| 33 | F | 4.13 | 11.5 | 34.6 | 83.7 | 24.3 | 26.8 | 13.5 | 26 | Hb E-trait | E |
| 35 | F | 5.23 | 12.7 | 38.2 | 73.1 | 23.4 | 28.3 | 13.6 | 29.4 | Hb E-trait | E |
| 26 | F | 4.53 | 11.0 | 33 | 72.8 | 25.5 | 27.3 | 13.2 | 29.2 | Hb E-trait | E |
| 30 | F | 4.68 | 11.0 | 33 | 70.5 | 23.2 | 26.4 | 13.5 | 26.2 | Hb E-trait | E |
| 38 | F | 4.44 | 11.7 | 35 | 78.8 | 23.6 | 28.4 | 14.1 | 29 | Hb E-trait | E |
| 31 | F | 5.68 | 11.3 | 34 | 59.9 | 21.2 | 23.1 | 13.5 | 14.3 | Hb E-trait | SEA/E |
| 23 | F | 4.47 | 11.3 | 34 | 76 | 25.3 | 33.2 | 12.6 | 26 | Hb E-trait | E |
| 19 | F | 4.38 | 11.1 | 33.3 | 76 | 25.5 | 28.2 | 14.2 | 29.1 | Hb E-trait | E |
| 28 | F | 4.74 | 12.0 | 36 | 76 | 23.6 | 26.4 | 13.6 | 27.8 | Hb E-trait | E |
| 19 | F | 4.27 | 11.7 | 35.2 | 82.4 | 25.3 | 28.3 | 14.2 | 26.4 | Hb E-trait | E |
| 38 | F | 4.87 | 12.0 | 36 | 73.9 | 27.8 | 29.8 | 12.8 | 28.3 | Hb E-trait | E |
| 27 | F | 4.19 | 11.0 | 33 | 78.8 | 23 | 25.8 | 14.2 | 25.9 | Hb E-trait | E |
| 38 | F | 4.63 | 12.7 | 38 | 82 | 25.3 | 28.1 | 13.2 | 27.6 | Hb E-trait | E |
| 23 | F | 5.31 | 12.7 | 38.2 | 73 | 25.6 | 28.3 | 13.6 | 27.4 | Hb E-trait | E |
| 25 | F | 5.34 | 12.0 | 36.1 | 67.6 | 22.4 | 24.5 | 14.2 | 20.9 | Hb E-trait | SEA/E |
| 43 | F | 4.27 | 11.7 | 35 | 82 | 25.3 | 28.4 | 12.6 | 27.1 | Hb E-trait | E |
| 22 | F | 4.41 | 11.7 | 35 | 79 | 26.5 | 33.2 | 12.8 | 29.7 | Hb E-trait | E |
| 28 | F | 4.81 | 12.3 | 37 | 77 | 25.3 | 27.8 | 13.2 | 27.5 | Hb E-trait | E |
| 30 | F | 4.43 | 11.7 | 35 | 79 | 25.4 | 27.3 | 13.5 | 29.6 | Hb E-trait | E |
| 33 | F | 4.93 | 12.0 | 36 | 73 | 24.3 | 26.8 | 13.5 | 27 | Hb E-trait | E |
| 35 | F | 4.02 | 11.0 | 33 | 82 | 23.6 | 26.2 | 12.8 | 27 | Hb E-trait | E |
| 23 | F | 5.67 | 11.7 | 35 | 61.7 | 23 | 26.3 | 13.6 | 19.7 | Hb E-trait | SEA/E |
| 31 | F | 4.26 | 11.0 | 33 | 77.5 | 20.1 | 22.6 | 14.2 | 25.9 | Hb E-trait | E |
| 33 | F | 4.93 | 12.0 | 36 | 73 | 20.3 | 23.1 | 13.2 | 21.8 | Hb E-trait | E |
| 43 | F | 4.27 | 11.7 | 35 | 82 | 25.3 | 28.4 | 12.6 | 27.1 | Hb E-trait | E |
| 22 | F | 4.41 | 11.7 | 35 | 79.3 | 26.5 | 33.2 | 12.8 | 29.7 | Hb E-trait | E |
| 28 | F | 4.81 | 12.3 | 37 | 77 | 25.3 | 27.8 | 13.2 | 27.5 | Hb E-trait | E |
| 28 | F | 5.51 | 12.7 | 38 | 69 | 23 | 25.9 | 13.5 | 19.3 | Hb E-trait | SEA/E |
| 30 | F | 4.43 | 11.7 | 35 | 79 | 25.4 | 27.3 | 13.5 | 29.6 | Hb E-trait | E |
| 33 | F | 4.93 | 12.0 | 36 | 73 | 24.3 | 26.8 | 13.5 | 27 | Hb E-trait | E |
| 35 | F | 4.02 | 11.0 | 33 | 82 | 23.6 | 26.2 | 12.8 | 27 | Hb E-trait | E |
| 33 | F | 4.93 | 12.0 | 36 | 73 | 20.3 | 23.1 | 13.2 | 21.8 | Hb E-trait | SEA/E |
| 33 | F | 4.93 | 12.0 | 36 | 73 | 24.3 | 26.8 | 13.5 | 27 | Hb E-trait | E |
| 35 | F | 4.02 | 11.0 | 33 | 82 | 23.6 | 26.2 | 12.8 | 27 | Hb E-trait | E |
| 25 | F | 5.67 | 11.7 | 35 | 61.7 | 23 | 26.3 | 13.6 | 15.8 | Hb E-trait | SEA/E |
| 28 | F | 4.71 | 13.0 | 39 | 82.8 | 25.3 | 28.4 | 14.2 | 27.9 | Hb E-trait | E |
| 43 | F | 4.27 | 11.7 | 35 | 82 | 25.3 | 28.4 | 12.6 | 27.1 | Hb E-trait | E |
| 22 | F | 4.41 | 11.7 | 35 | 79.3 | 26.5 | 33.2 | 12.8 | 25.6 | Hb E-trait | E |
| 39 | F | 4.13 | 11.5 | 34.6 | 83.7 | 24.3 | 26.8 | 13.5 | 26 | Hb E-trait | E |
| 42 | F | 4.41 | 11.7 | 35 | 79.3 | 26.5 | 33.2 | 12.8 | 30.9 | Hb E-trait | E |
| 29 | F | 3.97 | 9.9 | 29.7 | 75.6 | 24.6 | 32.5 | 14.5 | 29.1 | Hb E-trait | E |
| 22 | F | 4.41 | 11.7 | 35 | 79.3 | 26.5 | 33.2 | 12.8 | 29.7 | Hb E-trait | E |
| 24 | F | 4.67 | 12.1 | 36.4 | 78 | 25.3 | 27.8 | 13.2 | 28.3 | Hb E-trait | E |
| 28 | F | 5.51 | 12.7 | 38 | 69 | 23 | 25.9 | 13.5 | 19.3 | Hb E-trait | SEA/E |
| 33 | F | 4.43 | 11.7 | 35 | 79 | 25.4 | 27.3 | 13.5 | 29.6 | Hb E-trait | E |
| 30 | F | 4.93 | 12.0 | 36 | 73 | 24.3 | 26.8 | 13.5 | 27 | Hb E-trait | E |
| 35 | F | 4.41 | 11.0 | 33 | 75 | 23.6 | 26.2 | 12.8 | 27 | Hb E-trait | E |
| 25 | F | 5.67 | 11.7 | 35 | 61.7 | 23 | 26.3 | 13.6 | 19.7 | Hb E-trait | SEA/E |
| 24 | F | 5.38 | 11.7 | 35 | 65 | 24.5 | 26.8 | 13.6 | 19.6 | Hb E-trait | SEA/E |
| 32 | F | 4.21 | 10.7 | 32 | 76 | 25.3 | 28.4 | 14.1 | 31.7 | Hb E-trait | E |
| 30 | F | 4.38 | 11.0 | 33 | 75.4 | 25.5 | 28.3 | 13.5 | 26 | Hb E-trait | E |
| 24 | F | 5.13 | 13.4 | 40.2 | 78.3 | 26 | 26.3 | 13.6 | 26.4 | Hb E-trait | E |
| 29 | F | 4.75 | 12.7 | 38 | 80 | 24 | 26.4 | 13.2 | 30.1 | Hb E-trait | E |
| 28 | F | 4.50 | 11.7 | 35 | 77.7 | 24.9 | 26.8 | 13.6 | 31.1 | Hb E-trait | E |
| 19 | F | 4.38 | 11.1 | 33.3 | 76 | 25.5 | 28.2 | 14.2 | 29.1 | Hb E-trait | E |
| 26 | F | 4.28 | 12.0 | 36 | 84.2 | 27.7 | 26.4 | 13.6 | 25.2 | Hb E-trait | E |
| 38 | F | 4.61 | 12.3 | 37 | 80.3 | 23 | 26.1 | 12.4 | 29.5 | Hb E-trait | E |
| 36 | F | 4.32 | 12.3 | 36.8 | 85.1 | 24.8 | 26.3 | 12.8 | 26.2 | Hb E-trait | E |
| 33 | F | 4.13 | 11.5 | 34.6 | 83.7 | 24.3 | 26.8 | 13.5 | 26 | Hb E-trait | E |
| 38 | F | 4.44 | 11.7 | 35 | 78.8 | 23.6 | 28.4 | 14.1 | 29 | Hb E-trait | E |
| 23 | F | 4.47 | 11.3 | 34 | 76 | 25.3 | 33.2 | 12.6 | 26 | Hb E-trait | E |
| 19 | F | 4.38 | 11.1 | 33.3 | 76 | 25.5 | 28.2 | 14.2 | 29.1 | Hb E-trait | E |
| 28 | F | 4.74 | 12.0 | 36 | 76 | 23.6 | 26.4 | 13.6 | 27.8 | Hb E-trait | E |
| 30 | F | 4.27 | 11.7 | 35.2 | 82.4 | 25.3 | 28.3 | 14.2 | 26.4 | Hb E-trait | E |
| 29 | F | 4.32 | 10.7 | 32 | 74 | 23 | 26 | 13.2 | 61.2 | Hb E-trait | E |
| 19 | F | 4.38 | 11.1 | 33.3 | 76 | 25.5 | 28.2 | 14.2 | 29.1 | Hb E-trait | E |
| 28 | F | 4.50 | 12.0 | 36 | 80 | 23.6 | 26.4 | 13.6 | 27.8 | Hb E-trait | E |
| 29 | F | 4.32 | 10.7 | 32 | 76 | 23 | 26 | 13.2 | 30.6 | Hb E-trait | E |
| 19 | F | 4.38 | 11.1 | 33.3 | 76 | 25.5 | 28.2 | 14.2 | 28.1 | Hb E-trait | E |
| 37 | F | 4.41 | 11.7 | 35 | 79.3 | 26.5 | 33.2 | 12.8 | 30.9 | Hb E-trait | E |
| 34 | F | 4.07 | 10.7 | 32 | 78.6 | 24.3 | 26.8 | 12.8 | 28.4 | Hb E-trait | E |
| 26 | F | 4.53 | 11.0 | 33 | 72.8 | 25.5 | 27.3 | 13.2 | 29.2 | Hb E-trait | E |
| 30 | F | 4.68 | 11.0 | 33 | 70.5 | 23.2 | 26.4 | 13.5 | 26.2 | Hb E-trait | E |
| 38 | F | 4.44 | 11.7 | 35 | 78.8 | 23.6 | 28.4 | 14.1 | 29 | Hb E-trait | E |
| 31 | F | 5.68 | 11.3 | 34 | 59.9 | 21.2 | 23.1 | 13.5 | 15.8 | Hb E-trait | SEA/E |
| 37 | F | 4.41 | 11.7 | 35 | 79.3 | 26.5 | 33.2 | 12.8 | 30.9 | Hb E-trait | E |
| 22 | F | 4.21 | 11.9 | 35.6 | 85 | 28.2 | 33.2 | 12.4 | 29.6 | Hb E-trait | E |
| 34 | F | 4.07 | 10.7 | 32 | 78.6 | 24.3 | 26.8 | 12.8 | 22.6 | Hb E-trait | E |
| 26 | F | 4.53 | 11.0 | 33 | 72.8 | 25.5 | 27.3 | 13.2 | 29.2 | Hb E-trait | E |
| 24 | F | 4.67 | 11.7 | 35 | 75 | 24.6 | 26.4 | 13.6 | 26.5 | Hb E-trait | E |
| 25 | F | 4.68 | 11.0 | 33 | 70.5 | 23.2 | 26.4 | 13.5 | 26.2 | Hb E-trait | E |
| 31 | F | 5.68 | 11.3 | 34 | 59.9 | 21.2 | 23.1 | 13.5 | 18.6 | Hb E-trait | SEA/E |
| 23 | F | 4.47 | 11.3 | 34 | 76 | 25.3 | 33.2 | 12.6 | 26 | Hb E-trait | E |
| 19 | F | 4.38 | 11.1 | 33.3 | 76 | 25.5 | 28.2 | 14.2 | 29.1 | Hb E-trait | E |
| 23 | F | 4.87 | 12.0 | 36.1 | 74.1 | 24.6 | 33.2 | 13.5 | 29.9 | Hb E-trait | E |
