# Supplementary material for: Prevalence of double heterozygotes of HbE and α-thal 1 (SEA) type in pregnancies and their partners that received antenatal care at Chiangrai Prachanukroh Hospital and reevaluated the cut-offs for differentiation
Source: PLoS One. 2025 Oct 7;20(10):e0333761. doi: 10.1371/journal.pone.0333761 (PMC12503262; doi:10.1371/journal.pone.0333761)
Supplement: Attachment — Submitted filename: Comments for PLoS ONE.docx [file pone.0333761.s006.docx]

**Comments-**

In this paper, Leckngam P, et al describe their study on the hematological and hemoglobin analysis, comparing pure Hb E heterozygote and double heterozygote for Hb E and α-thalassemia 1. Various hematological parameters were described and compared to differentiate the two groups. Study was done retrospectively on selected group of subjects with specified criterions. Unfortunately, the finding is not new to warrant publication. In fact, differentiation of the two groups of Hb E could be made easily using just the level of Hb E (+Hb A_2_) on Hb analysis. A screening protocol regarding this has been well established and described in the national guideline, being applied throughout the country with high prevalence of thalassemia like Thailand. Other criticisms are listed below.

1. The term “double heterozygotes SEA/E” used throughout the whole manuscript is not suitable especially in the title which should be self-explainable. I believe that readers outside the field of hemoglobinopathy study would not understand this term.
2. As for no. 1, the term “single Hb E heterozygote” is not acceptable. This could mean a single copy of Hb E as seen in heterozygote, but in a double heterozygote SEA/E described in the manuscript, you also have a single copy of Hb E. This could be confusing. I do understand that the authors use the term “single Hb E heterozygote” to describe a pure Hb E heterozygote (i.e., without α-thalassemia). This could be simply read as Hb E heterozygote.
3. Introduction part is too long, containing several unnecessary information,… could be reduced by half. In addition, I do not understand the meaning of the last sentence of the introduction. … “It’s at this hospital that pregnant women attend very much, which accounts for approximately 15% of all pregnancies in Chiangrai province”… Re-writing is needed.
4. It has been known for years that Hb E heterozygote with Hb E < 25% could be co-inherited with α -thalassemia and further DNA analysis of α-thalassemia 1 is recommended. This has been accepted in the general guideline of thalassemia screening in many areas including Thailand. In fact, you have also seen this in your study as shown in the Table 2 of the current paper (28.1±2.8 v.s. 18.9±3.4 and also shown in Figure 2D). This is a very simple differentiation parameter available after Hb analysis. No need to look for any other complicated cut-off values as described in the current paper. The screening protocol regarding this has been published for years in the two papers (Fucharoen G, et al. A simplified screening strategy for thalassaemia and haemoglobin E in rural communities of Southeast Asia. *Bulletin World Health Organ 2004; 82: 364-372.* and Sanchaisuriya K, et al. A reliable screening protocol for thalassemia and hemoglobinopathies in pregnancy; an alternative approach to electronic blood cell counting. *Am J Clin Pathol 2005; 123: 113-118*.). Unfortunately, these two papers have not been mentioned in the current paper by Leckngam P, et al.
5. Subjects were selectively and retrospectively recruited under specified criterion. They could be regarded as known samples. With this approach, it is not possible to calculate accurate sensitivity, specificity, NPV and PPV. The authors may use the data of these subjects to establish the cut-off value but they need to prove it prospectively on unknown subjects recruited continuously in independent cohorts (at least three is best) before reliable analytical characteristics can be calculated.
6. Table 1 is not necessary. As for Figure 1 which describes well known knowledge of genetic inheritance,… this is no need….

With the above criticisms and minute novel information, I do not think that this paper is suitable for publication in PLoS ONE.
