# Supplementary material for: Prevalence of double heterozygotes of HbE and α-thal 1 (SEA) type in pregnancies and their partners that received antenatal care at Chiangrai Prachanukroh Hospital and reevaluated the cut-offs for differentiation
Source: PLoS One. 2025 Oct 7;20(10):e0333761. doi: 10.1371/journal.pone.0333761 (PMC12503262; doi:10.1371/journal.pone.0333761)
Supplement: Attachment — Submitted filename: Response to Reviewers.docx [file pone.0333761.s008.docx]

**Response to reviewers**

PONE-D-25-05285

Prevalence of double heterozygotes SEA/E in pregnancies and their couples that received Antenatal Care at Chiangrai Prachanukroh Hospital

PLOS ONE

Please include the following items when submitting your revised manuscript:

- A rebuttal letter that responds to each point raised by the academic editor and reviewer(s). You should upload this letter as a separate file labeled 'Response to Reviewers'.
- A marked-up copy of your manuscript that highlights changes made to the original version. You should upload this as a separate file labeled 'Revised Manuscript with Track Changes'.
- An unmarked version of your revised paper without tracked changes. You should upload this as a separate file labeled 'Manuscript'.

**Journal Requirements:**

1. Please state what role the funders took in the study.  If the funders had no role, please state: "The funders had no role in study design, data collection and analysis, decision to publish, or preparation of the manuscript."

- Answer….Mae Fah Luang University, the funder, had no role in the study design, data collection and analysis, decision to publish or preparation of the manuscript.

2. Please include this amended Role of Funder statement in your cover letter; we will change the online submission form on your behalf.

Answer……It has been added in cover letter.

3. Thank you for stating the following in the Acknowledgments Section of your manuscript: I would like to express gratitude to the director of Chiangrai Prachanukroh Hospital for the opportunity to conduct this research and to the head of the Antenatal Care Clinic and the nursing team in the hospital for dedicating their time to collect data. I also thank my advisor for consultation and for giving valuable information to this research. Thanks also to Mae Fah Luang University for funding this research. We note that you have provided funding information that is not currently declared in your Funding Statement. However, funding information should not appear in the Acknowledgments section or other areas of your manuscript. We will only publish funding information present in the Funding Statement section of the online submission form.

- Please remove any funding-related text from the manuscript and let us know how you would like to update your Funding Statement. Currently, your Funding Statement reads as follows: Mae Fah Luang University, Grant no. 651A05003
- Answer….I have removed funding information from Acknowledgement section and other areas of the manuscript.

4. One of the noted authors is a group or consortium. In addition to naming the author group, please list the individual authors and affiliations within this group in the acknowledgments section of your manuscript. Please also indicate clearly a lead author for this group along with a contact email address.

- Answer…..We have made the corrections.

5. Your ethics statement should only appear in the Methods section of your manuscript. If your ethics statement is written in any section besides the Methods, please delete it from any other section.

- Answer…..We have made the corrections.

6. Please include captions for your Supporting Information files at the end of your manuscript, and update any in-text citations to match accordingly. Please see our Supporting Information guidelines for more information: <http://journals.plos.org/plosone/s/supporting-information>.

- Answer…. We have made the corrections.

**Additional Editor Comments:**
I have read the manuscript in detail and appreciate the effort that has gone into the study. I am pleased to note that two reviewers have recommended revision; however, the third reviewer has expressed reservations about publishing the manuscript in PLOS ONE. I trust the authors are willing to revise the manuscript in line with the reviewers’ comments.

7. The inclusion of couples carrying double heterozygous mutations for HbE and SEA α-thalassemia presents a novel aspect of the study. However, unless the recruitment of couples is clearly justified, the analysis may appear to reflect only individual-level screening. It seems the authors have established substantial rapport with the participating couples, which likely included genetic counselling. If available, pregnancy outcomes from these couples would significantly enrich the dataset and add a novel dimension to the findings.

- Answer……Based on data collected over the 5 past years, from 2017 to 2021, there have been no partners at-risk who both carry double SEA/E or one who carries double SEA/E and another carries heterozygote of α-thal 1 (SEA) type giving a fetus with Hb Bart’s hydrops fetalis. However, if there is a partner at-risk, to allow this partner to independently decide which method to choose to avert and prevent having a child with the disease. The goal is to empower this partner to make a decision on the best method to prevent the birth of a child with this disease. The key issue is selecting the preferred prenatal diagnosis method and planning for severe disease results, such as terminating the pregnancy. We have added.

8. I encourage the authors to further highlight the novelty of their work. Since contact with participants has already been established, incorporating additional socio-economic variables would enhance the analytical depth of the study.

- The novelty of this study is that it excludes samples that may be contaminated with iron-deficiency anemia by using RDW cut-off value of more than 14.5% (good indicator and low costs) (Polprasert et al., 2020) that helps to reduce false positive screening. This research helps to reduce the occurrence of Hb Bath’s hydrops fetalis. Additionally, it aids in reducing socio-economic problem for families and lowers the country’s health care costs.
- Answer ……It has been added.

9. The manuscript, submitted in February 2025, includes data from individuals recruited between 2017 and 2021. Given this timeline, follow-up records are expected to be available. Since couple-based recruitment is central to the study, I recommend including all available follow-up data to better capture outcomes such as pregnancy loss or neonatal complications—this will considerably strengthen the paper.

- Answer…. Based on data collected over the 5 past years, from 2017 to 2021, there have been no cases of partners who both carry double SEA/E or one who carries double SEA/E and another carries heterozygote of α-thal 1 (SEA) type giving a fetus with Hb Bart’s hydrops fetalis. However, if there is a partner at-risk, to allow this partner to independently decide which method to choose to avert and prevent having a child with the disease. The goal is to empower this partner to make a decision on the best method to prevent the birth of a child with this disease. The key issue is selecting the preferred prenatal diagnosis method and planning for severe disease results, such as terminating the pregnancy.
- Answer ……It has been added.

10. Table 1 currently presents data for 397 individuals but lacks couple-based information, which is essential considering the study's focus. Please add a cross-tabulation showing different combinations of hemoglobin variants among couples. Also, the age-related distributions are somewhat inconsistent and should be corrected for clarity.

- Answer……Because the data is collected in a personal manner and not in pairs, there is no information in this section. Age-related distributions have been corrected as in Table 1.

11. Table 2 compares RBC parameters between single HbE heterozygotes and SEA/E double heterozygotes but does not stratify by sex, which is clinically important.

- Answer……It has been added in Table 3.

12. Table 3 should be revised to present sex-specific data.

- Answer…….Because in Thailand, cut-off values are used to separate HbE heterozygote from double heterozygotes of HbE and α-thal 1 (SEA) type without gender distinction; therefore, I would like to keep the data in this section as it is.

13. In Lines 259–260, the statement “50 double heterozygotes SEA/E were found among 397 individuals (26 pregnant women and 24 male partners)” is unclear and should be clarified.

- Answer…..It has been corrected.

14. A map of the study area should be included to provide geographic context.

- Answer …It has been added in Fig 3.

15. The sentence in Line 292, “validation of cut-offs classified according to hemoglobin concentrations was performed,” should be revised to better reflect the updated analysis and inferences.

- Answer…….It has been corrected.

16. The supplementary tables should be updated to reflect couple-level (partnered) information rather than individual data.

- Answer……Because the data is collected in a personal manner and not in pairs, there is no information in this section.

17. Finally, the title should be reconsidered to better reflect the focus and novelty of the study, in light of the reviewers' comments.

- Answer….change the title to “Prevalence of double heterozygotes of HbE and α-thal 1 (SEA) type in pregnancies and their partners that received Antenatal Care at Chiangrai Prachanukroh Hospital and reevaluated the cut-offs for differentiation in samples that excluded iron deficiency anemia”

I am appending the reviewers’ comments for your kind attention and necessary action.

**Reviewer 1**
Regarding Question 1. Technical Soundness and Data Support for Conclusions
The manuscript presents a well-conducted study with a sound methodology and appropriate data collection techniques. The data support the conclusions drawn, particularly in terms of the prevalence of SEA/E double heterozygotes and the use of hematological parameters for differentiation. However, the study would benefit from a more explicit discussion of potential confounding variables, such as iron deficiency anemia, which may affect MCV and MCH values.

**Recommendation:**
1. Include a discussion on how confounding variables, such as iron deficiency, may have influenced the hematological parameters in the discussion section.

Answer….Due to iron-deficiency anemia, MCV and MCH are lower similar to thalassemia trait. To reduce false positive screening if there are contaminated in samples. RDW >14.5% is a good indicator and can help to excluded samples with iron-deficiency anemia at no additional costs. It has been added.

2. Statistical Analysis
The statistical tests are appropriately applied. However, additional justification for the selected p-value threshold of 0.05 would improve transparency.

- Answer……Because the parameters that were compared included in diagnostic tests.

- It has been added.

Recommendation:
Provide a brief explanation for choosing p<0.05 as the statistical significance threshold in the Methods section, after line 172.

- Answer…..It has been corrected.

3. Data Availability
The manuscript states that “all relevant data are within the manuscript and its supporting information files,” but no publicly accessible dataset is provided.
While ethical concerns may restrict sharing, authors should clarify whether anonymized data can be made available upon request.

-	Answer …All data are in the supplement Table 2.

1. Recommendation:
   Indicate whether anonymized datasets or raw data will be deposited in a public repository, or provide a justification for restrictions.

- Answer …All data are in the supplement Table 2.
- Notable Errors are:

1. Line 23: “If pregnant women who carry this genotype get pregnant with their partners…” → Suggested revision: “If pregnant women carrying this genotype conceive with partners…”

-	Answer…..It has been corrected.

2. Line 96: “Due to the poor survival of an affected fetuses…”
   → Suggested revision: “Due to the poor survival rate of affected fetuses…”

- Answer…..It has been corrected.

3. Line 125: “It’s at this hospital that pregnant women attend very much…”
   → Suggested revision: “This hospital serves a high volume of pregnant women…”

- Answer…..It has been corrected.

4. Line 280: “Our study is similar to the large cohort study from Charoenkwan P. et al. (2005)…” → Suggested revision: “Our study is similar to the large cohort study by Charoenkwan et al. (2005)…”

- Answer…..It has been corrected.

5. Additional Recommendations.

- Quality Control Measures: Explain how quality control was ensured in laboratory procedures for HPLC and PCR testing in the method section, after line 164.
- Answer…..It has been added.

6. Conclusion Expansion: Provide more actionable recommendations on how these findings could influence policy and clinical guidelines in the conclusion section, after line 312.

- Answer…..It has been added.

7. Significance and Validity of Claim: The authors claim that appropriate cut-offs for hematological parameters and HbE levels can distinguish SEA/E carriers from single HbE heterozygotes. While the claims are valid, a stronger comparison with similar studies in other populations would improve the generalizability of the findings.

- Answer…..It has been added.

**Reviewer 2**
The manuscript examines the "Prevalence of double heterozygotes SEA/E in pregnancies and their couples who received antenatal care at Chiangrai Prachanukroh Hospital." This research is crucial for identifying pregnant women affected by this genetic condition, as it can play a significant role in allowing healthcare professionals to implement targeted strategies that could help prevent or effectively manage various complications. These complications may extend beyond physical health to include psychological and mental health challenges. However, the findings and conclusions outlined in the manuscript fall short of establishing clear clinical correlations. There is also a noticeable absence of practical recommendations to guide healthcare providers on how to support these women by utilizing these effective screening measures and management options. It is essential that this vital information be incorporated into both the discussion and the conclusion sections of the manuscript to enhance its practical applicability and relevance for clinical practice.
Answer…It has been corrected.

2. Title
The study's title, "Prevalence of Double Heterozygotes SEA/E in Pregnancies and Their Couples Who Received Antenatal Care at Chiangrai Prachanukroh Hospital," is confusing. Did the authors intend to use "partners" instead of "couples" in the title? The term "couples" is unclear in this context, and it would be better to clarify what the authors are referring to.

Answer … It has been corrected.

3. Introduction
Page 4, lines 68-70: The statement seems incomplete with the word "who". Kindly review and make this statement more clearer.

Answer…..It has been corrected.

4. Page 5, line 93: There should be a reference to support the figure quoted in the sentence.

Answer… It has been added in line 91, Fig 1.

5. Page 5, lines 94-96: There should also be a reference to support the statement made on the risk of perinatal death and associated complications for the mother.

Answer…..It has been added line 93.

6. Page 6, lines 123-126: The statement made here should be under the method section. The authors should review this make appropriate correction.

- Answer…..It has been moved to the method section.

7. Page 6, lines 128-130: The figure stated here, Figure 1, should be closer to its first mention. Currently, readers need to search for where the figure is referenced in the manuscript.

Answer…..It has been corrected.

Methods
8. Page 6, line 134. The authors should clarify the earlier comments regarding the title, which is also reflected here, particularly concerning "pregnant women and their couples." Do the authors mean "partners" instead? It is important to clarify this for better understanding.

- Answer …The correction involved using the word ‘partners’ instead of ‘couples’.

9. Page 8, lines 178-179: The authors should provide a detailed explanation of the significance of critical parameters, including sensitivity, specificity, positive predictive value (PPV), negative predictive value (NPV), and overall accuracy. Additionally, they need to establish precise grade levels to interpret these metrics accurately. It would be beneficial to specify the thresholds that classify these parameters as low, moderate, and high, enabling readers to understand the implications of varying results more effectively.

Answer….It has been added in discussion.

10. Page 10, lines 206-208: The statement made under the validation of cutoffs used should be in the method section. The result section should solely focus on the study's findings.

Answer… It has been corrected.

11. Page 210-222: It is essential to define the metrics used for validating the cutoffs in the methods section to enhance clarity and help readers understand their significance in the validation process. Additionally, it is important to present the classification levels of these tests—such as low, moderate, or high—so that readers can relate to them more effectively.

Page 11, lines 227-232, For Tables 1 and 2, the authors should define all abbreviations as footnotes under the table.

Answer….. It has been corrected.

12. Page 12 and 13: The authors should define the abbreviations on Tables 3 and 4 as well as footnote.

- Answer…..It has been added.

13. Discussion:
Page 14, lines 264-266: The authors pointed out that mothers affected by this genetic disorder may experience some complications, yet they did not detail what those specific complications might entail. Identifying these complications is essential for proper understanding and management. Furthermore, the authors should elucidate how these complications can be prevented, drawing on the findings of this study. This knowledge is vital for implementing these screening measures that can either avert these complications entirely or ensure they are managed accurately and efficiently when they do occur.

Pages 13-15, lines 254-299: The discussion lacks depth in interpreting this study's findings and seems to merely reiterate the results. Additionally, there is insufficient clinical correlation to these findings. For instance, what are the clinical implications of these results in real-world settings? How might they impact practice, research, and policy? In what ways can they enhance overall clinical practice? Moreover, is there a need for future research in this area? If so, which specific aspects should be explored further? Therefore, the discussion should be refined to integrate these findings better.

Answer…..The limitation of the research is that it did not test for serum iron or ferritin to investigate for iron deficiency anemia.

14. Conclusion:
Page 16, lines 310-312: The basis for the recommendation made as well as the recommendation itself did not reflect in the discussion.

Acknowledgment
Page 16, line 315: I believe the manuscript write-up is a joint effort between the authors. Consequently, the opening statement that begins with "I would like to express" fails to capture the essence of this teamwork and the collective dedication to the study. Given the involvement of other authors, it is essential to refrain from using first-person language, as it does not reflect the unified contributions made by the team. Instead, employing third-person language will more accurately highlight the shared insights, perspectives, and expertise of all contributors, underscoring the spirit of collaboration that underpins this work.

Answer…..It has been corrected.

**Reviewer 3**
In this paper, describe their study on the hematological and hemoglobin analysis, comparing pure Hb E heterozygote and double heterozygote for Hb E and α-thalassemia 1. Various hematological parameters were described and compared to differentiate the two groups. Study was done retrospectively on selected group of subjects with specified criterions. Unfortunately, the finding is not new to warrant publication. In fact, differentiation of the two groups of Hb E could be made easily using just the level of Hb E (+Hb A2) on Hb analysis. A screening protocol regarding this has been well established and described in the national guideline, being applied throughout the country with high prevalence of thalassemia like Thailand. Other criticisms are listed below.
1. The term “double heterozygotes SEA/E” used throughout the whole manuscript is not suitable especially in the title which should be self-explainable. I believe that readers outside the field of hemoglobinopathy study would not understand this term.

Answer……..Change to double heterozygotes of HbE and α-thal 1 (SEA) type.

2. As for no. 1, the term “single Hb E heterozygote” is not acceptable. This could mean a single copy of Hb E as seen in heterozygote, but in a double heterozygote SEA/E described in the manuscript, you also have a single copy of Hb E. This could be confusing. I do understand that the authors use the term “single Hb E heterozygote” to describe a pure Hb E heterozygote (i.e., without α-thalassemia). This could be simply read as Hb E heterozygote.

Answer…….change single HbE heterozygote to pure HbE heterozygote

3. Introduction part is too long, containing several unnecessary information,… could be reduced by half. In addition, I do not understand the meaning of the last sentence of the introduction. … “It’s at this hospital that pregnant women attend very much, which accounts for approximately 15% of all pregnancies in Chiangrai province”… Re-writing is needed.

Answer…..We have removed the unnecessary content and re-writing.

4. It has been known for years that Hb E heterozygote with Hb E < 25% could be co-inherited with α -thalassemia and further DNA analysis of α-thalassemia 1 is recommended. This has been accepted in the general guideline of thalassemia screening in many areas including Thailand. In fact, you have also seen this in your study as shown in the Table 2 of the current paper (28.1+2.8 v.s. 18.9+3.4 and also shown in Figure 2D). This is a very simple differentiation parameter available after Hb analysis. No need to look for any other complicated cut-off values as described in the current paper. The screening protocol regarding this has been published for years in the two papers (Fucharoen G, et al. A simplified screening strategy for thalassaemia and haemoglobin E in rural communities of Southeast Asia. Bulletin World Health Organ 2004; 82: 364-372. and Sanchaisuriya K, et al. A reliable screening protocol for thalassemia and hemoglobinopathies in pregnancy; an alternative approach to electronic blood cell counting. Am J Clin Pathol 2005; 123: 113-118.). Unfortunately, these two papers have not been mentioned in the current paper.

Answer……The new concept of this research is to re-evaluate cut-offs based on hemoglobin levels and excluded samples with iron-deficiency anemia.

5. Subjects were selectively and retrospectively recruited under specified criterion. They could be regarded as known samples. With this approach, it is not possible to calculate accurate sensitivity, specificity, NPV and PPV. The authors may use the data of these subjects to establish the cut-off value but they need to prove it prospectively on unknown subjects recruited continuously in independent cohorts (at least three is best) before reliable analytical characteristics can be calculated.

Answer…The objective of this research is to reevaluate cut-offs classified according hemoglobin levels in samples that excluded iron-deficiency anemia by using RDW >14.5% according to the study of Polprasert et al., 2020. This helps minimise false positive screening.

6. Table 1 is not necessary. As for Figure 1 which describes well known knowledge of genetic inheritance,… this is no need…. With the above criticisms and minute novel information, I do not think that this paper is suitable for publication in PLoS ONE.

Answer… It has been improved appropriately.

**Reviewer #3:** In this paper, Leckngam P, et al describe their study on the hematological and hemoglobin analysis, comparing pure Hb E heterozygote and double heterozygote for Hb E and α-thalassemia 1. Various hematological parameters were described and compared to differentiate the two groups. Study was done retrospectively on selected group of subjects with specified criterions. Unfortunately, the finding is not new to warrant publication. In fact, differentiation of the two groups of Hb E could be made easily using just the level of Hb E (+Hb A2) on Hb analysis. A screening protocol regarding this has been well established and described in the national guideline, being applied throughout the country with high prevalence of thalassemia like Thailand. Other criticisms are listed below.

1. The term “double heterozygotes SEA/E” used throughout the whole manuscript is not suitable especially in the title which should be self-explainable. I believe that readers outside the field of hemoglobinopathy study would not understand this term.

2. As for no. 1, the term “single Hb E heterozygote” is not acceptable. This could mean a single copy of Hb E as seen in heterozygote, but in a double heterozygote SEA/E described in the manuscript, you also have a single copy of Hb E. This could be confusing. I do understand that the authors use the term “single Hb E heterozygote” to describe a pure Hb E heterozygote (i.e., without α-thalassemia). This could be simply read as Hb E heterozygote.
3. Introduction part is too long, containing several unnecessary information,… could be reduced by half. In addition, I do not understand the meaning of the last sentence of the introduction. … “It’s at this hospital that pregnant women attend very much, which accounts for approximately 15% of all pregnancies in Chiangrai province”… Re-writing is needed.

Answer….It has been re-writing.

4. It has been known for years that Hb E heterozygote with Hb E < 25% could be co-inherited with α -thalassemia and further DNA analysis of α-thalassemia 1 is recommended. This has been accepted in the general guideline of thalassemia screening in many areas including Thailand. In fact, you have also seen this in your study as shown in the Table 2 of the current paper (28.1+2.8 v.s. 18.9+3.4 and also shown in Figure 2D). This is a very simple differentiation parameter available after Hb analysis. No need to look for any other complicated cut-off values as described in the current paper. The screening protocol regarding this has been published for years in the two papers (Fucharoen G, et al. A simplified screening strategy for thalassaemia and haemoglobin E in rural communities of Southeast Asia. Bulletin World Health Organ 2004; 82: 364-372. and Sanchaisuriya K, et al. A reliable screening protocol for thalassemia and hemoglobinopathies in pregnancy; an alternative approach to electronic blood cell counting. Am J Clin Pathol 2005; 123: 113-118.). Unfortunately, these two papers have not been mentioned in the current paper by Leckngam.

5. Subjects were selectively and retrospectively recruited under specified criterion. They could be regarded as known samples. With this approach, it is not possible to calculate accurate sensitivity, specificity, NPV and PPV. The authors may use the data of these subjects to establish the cut-off value but they need to prove it prospectively on unknown subjects recruited continuously in independent cohorts (at least three is best) before reliable analytical characteristics can be calculated.

6. Table 1 is not necessary. As for Figure 1 which describes well known knowledge of genetic inheritance,… this is no need….

Answer… It has been improved appropriately.
