# Supplementary material for: Prevalence of double heterozygotes of HbE and α-thal 1 (SEA) type in pregnancies and their partners that received antenatal care at Chiangrai Prachanukroh Hospital and reevaluated the cut-offs for differentiation
Source: PLoS One. 2025 Oct 7;20(10):e0333761. doi: 10.1371/journal.pone.0333761 (PMC12503262; doi:10.1371/journal.pone.0333761)
Supplement: Attachment — Submitted filename: Response to Reviewers..docx [file pone.0333761.s009.docx]

**Response to reviewers**

PONE-D-25-05285R1

Prevalence of double heterozygotes of HbE and α-thal 1 (SEA) type in pregnancies and their partners that received Antenatal Care at Chiangrai Prachanukroh Hospital and reevaluated the cut-offs for differentiation

**Please include the following items when submitting your revised manuscript:**

- A rebuttal letter that responds to each point raised by the academic editor and reviewer(s). You should upload this letter as a separate file labeled 'Response to Reviewers'.
- A marked-up copy of your manuscript that highlights changes made to the original version. You should upload this as a separate file labeled 'Revised Manuscript with Track Changes'.
- An unmarked version of your revised paper without tracked changes. You should upload this as a separate file labeled 'Manuscript'.

**Journal Requirements:**

Please review your reference list to ensure that it is complete and correct. If you have cited papers that have been retracted, please include the rationale for doing so in the manuscript text, or remove these references and replace them with relevant current references. Any changes to the reference list should be mentioned in the rebuttal letter that accompanies your revised manuscript. If you need to cite a retracted article, indicate the article’s retracted status in the References list and also include a citation and full reference for the retraction notice.

Answer ….. In the second revision, the references have been reordered due to images being added. Line 512-550.

**Additional editor comments**

As the original reviewers were unavailable to confirm their acceptance, a second round of peer review was conducted, which yielded a positive outcome. I kindly request you to revise your manuscript for a final evaluation, after which it may be considered for publication. Additionally, I urge you to carefully revise the English language with the help of professional editorial support.

**Review comments to the author**

Reviewer #4: The manuscript offers a compelling and methodologically sound contribution to the field of prenatal genetic screening, particularly in the context of thalassemia control in northern Thailand. The study addresses an important public health issue by evaluating the diagnostic accuracy of hematological parameters in distinguishing HbE heterozygotes from compound heterozygotes of HbE and α-thalassemia 1 (SEA type), which has significant implications for the prevention of severe conditions such as Hb Bart’s hydrops fetalis. A major strength of the study is the careful exclusion of subjects with elevated red cell distribution width (RDW), effectively controlling for the confounding effect of iron deficiency anemia. This step strengthens the internal validity of the findings and reflects careful attention to potential biases. The use of adjusted hematological cut-off values is particularly noteworthy. These refined parameters improve diagnostic specificity and sensitivity, enhancing the potential utility of routine blood indices as a frontline screening method in resource-limited settings. The authors’ conclusions are well-supported by the data and align with broader goals in reproductive health and genetic counseling in high-prevalence-regions.

**Areas for improvement**

Language and Grammar: Some sections contain verbose or awkward sentence constructions. A professional language edit is recommended to improve fluency and precision. Sentences are too long or wordy, often using more words than necessary. This can make the meaning unclear or tiring for the reader. Example (Verbose): “The data which was collected over a period of ten years was thoroughly analyzed using a number of statistical methods which included…”
Improved: “Data collected over ten years were analyzed using multiple statistical methods…”
Phrases or sentences that may sound unnatural, unclear, or grammatically incorrect, even the meaning somewhat is understandable.

Example: “The outcomes are according to what was expected due to the previous findings.”
Improved: “The outcomes align with previous findings.”

2.Several abbreviations (e.g., MCV, MCH, RDW, Hb Bart’s) are used extensively. Ensure each is clearly defined on first use and consistently applied thereafter to avoid redundancy and confusion.
Figures and Supplementary Material: All figures and supplementary tables must be referenced accurately within the main text. Their accessibility and interpretability should be ensured, especially if they form part of the data validation.

Answer ….. It has been examined that abbreviations are clearly defined (lines 99-101 and 176) and the references have been reordered. All figures, tables and supplements have been verified accurately.

3.Clinical Context: Consider briefly expanding on the practical implications of these findings for frontline health workers or local healthcare systems. This would contextualize the results for implementation.

Answer…The practical impact of this study is that it allows the medical laboratory personnel to distinguish pure HbE heterozygote from double heterozygotes of HbE and α-thal 1 (SEA) type more accurately and effectively, lines 379-381.

Reviewer #5: The paper is well framed highlighting the prevalence of double heterozygotes of HbE and α-thal 1 (SEA) among antenatal women in Thailand which plays a key role in identifying couples with this genotype and offering genetic counselling to reduce the risk of bearing an affected child. The authors made an effort to stratify the hematological parameters to differentiate between the pure HbE heterozygotes and double heterozygotes of HbE and α-thal 1 (SEA) which is cost and time effective. Inclusion of RDW to rule out iron deficiency anemia is a good indicator. However, the authors should have evaluated this including serum ferritin which they considered as their limitation. The authors excluded iron deficiency anemia in their samples which adds novelty to the paper.

**Recommendations and Revisions suggested:**
1. Has the author sought informed consent from the 397 individuals enrolled in the study for screening? If so, include the statement of informed consent from the antenatal couples enrolled in the study under methodology section.

Answer….The study received ethical approval from the Human Research Ethics Committee of Chiangrai Prachanukroh Hospital on February 20, 2023 (EC CRH 006/66 Ex). It has been exempted from the informed consent due to its being a retrospective study based on medical records. Has been added in the methodology section, line 139.

2. Include the statistics used to determine the cut offs for MCV, MCH and HbE levels in the methodology section.

Answer….Receiver Operation Curve (ROC) analysis was performed to determine the optimal cutoff values. Has been added in the methodology section line 184-185.

3. In section Discussion, the authors have determined the single and combined cut offs in three different groups based on Hb levels. But it would be better if they explain why MCH + HBE cut offs were considered for Hb<10g/dl grp, MCV+MCH+HbE were considered for Hb-10-11.9 and MCV+HbE for Hb>12g/dl. Also specify how these cut offs can be considered as significant markers in differentiating pure HbE heterozygotes from double heterozygotes of HbE and α-thal 1 (SEA).

Answer ……Reasons for considering cut-offs have been added in the discussion section, lines 366-369.

Minor Revisions: As per Revised Manuscript
In Line 38, replace expand the term double SAE/E

- Answer…..It has been corrected. line 38-39.

In Line 39, replace the term reevaluated with reevaluation

- Answer…..It has been corrected. line 40.
- In Line 82, replace the word ethic to ethnic
- Answer…..It has been corrected. line 83.

In Line 82, sentence” in a studied by Tatu T et al” should be corrected as “In a study by…….”

- Answer…..It has been corrected, line 83.

In Line 131, reframe the sentence “This retrospective study …………records.

- Answer…..It has been reframed. Line 132

In Line 179 and 257 replace the term Reevaluated to Reevaluation

- Answer…..It has been corrected line 181 and 260.

In Line 271, check the statement Similar to the results obtained when using MCV. Clarify whether it is MCV or MCH.

- Answer…..It has been checked. It is MCV, line 274
